# Investigation on the regulatory effects of kaempferol on immune, inflammatory, and antioxidant functions in late laying hens

**DOI:** 10.3389/fvets.2026.1763833

**Published:** 2026-04-13

**Authors:** Xinyue Fu, Yan Liu, Leqi Tong, Jie Zhang, Shaolong Zhuang, Yuyun Gao, Jing Zhang

**Affiliations:** 1 Department of Animal Science, College of Animal Science, Fujian Agriculture and Forestry University, Fuzhou, China; 2 Fujian Hexing Ecological Agriculture Science and Technology Co., Ltd., Quanzhou, China

**Keywords:** antioxidant function, immune-inflammatory balance, kaempferol, late-laying hens, lipid metabolism

## Abstract

This study was conducted to systematically evaluate the regulatory effects of kaempferol (KAE) on lipid metabolism, immune function, inflammatory responses, and antioxidant capacity in late laying hens. A total of 288 49-week-old Jinghong No. 1 laying hens with uniform body weight and consistent laying performance were randomly assigned to four treatment groups, with six replicates per group and 12 hens per replicate. Following a 1-week acclimation period, a formal feeding trial was conducted for 10 weeks. The control group (CON) was fed a basal corn-soybean meal diet, while Experimental Groups I, II, and III received the same basal diet supplemented with 0.2, 0.4, and 0.6 g/kg KAE, respectively. In terms of lipid metabolism, compared with the CON group, dietary KAE supplementation in Groups I, II, and III significantly reduced hepatic vacuole area, with the most pronounced effect observed in Group II; additionally, Groups II and III had significantly lower serum triglyceride (TG) levels and egg yolk total cholesterol content (*p* < 0.05). For antioxidant function, Groups II and III exhibited a significant increase in serum glutathione peroxidase (GSH-PX) activity relative to the CON group (*p* < 0.05); Group II showed a tendency toward higher hepatic total antioxidant capacity (T-AOC) activity (*p* = 0.072) and elevated relative expression of superoxide dismutase 2 (*SOD2*) in uterine tissue (*p* = 0.07). Furthermore, Groups II and III displayed a significant upregulation in the relative expression of superoxide dismutase 1 (*SOD1*) in ovarian tissue (*p* < 0.05). With respect to inflammatory cytokines and humoral immune parameters, Groups II and III had significantly higher serum interleukin-4 (IL-4) levels and significantly lower serum interleukin-1β (IL-1β) levels compared with the CON group (*p* < 0.05), whereas no significant differences in serum immunoglobulin levels (IgM, IgG, IgA) were found between any of the KAE-supplemented groups and the CON group (*p* > 0.05). In ovarian tissue, Groups II and III showed a significant decrease in the relative expression of *IL-1*β and a significant increase in the relative expression of *IL-4* and interleukin-10 (*IL-10*) (*p* < 0.05). In uterine tissue, Groups II and III exhibited a significant reduction in the relative expression of *IL-1*β and interleukin-6 (*IL-6*), as well as a significant increase in the relative expression of *IL-4* and *IL-10* (*p* < 0.05). In conclusion, dietary kaempferol supplementation can ameliorate hepatic lipid deposition, enhance antioxidant capacity, and modulate the immune-inflammatory balance in late laying hens, thereby effectively alleviating the physiological decline associated with the late laying period.

## Graphical Abstract

**Figure:**
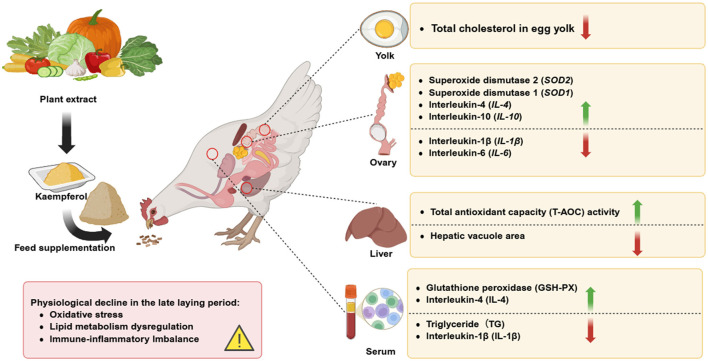

## 1 Introduction

Layer farming is a crucial pillar of the global poultry industry, providing humans with a stable and high-quality source of animal protein (1). During the late laying period, laying hens typically enter a subhealthy state due to age-related physiological decline and prolonged laying stress, which is accompanied by disrupted metabolic homeostasis, excessive production of reactive oxygen species (ROS), and impaired resistance to oxidative stress. Simultaneously, damage to immunocompetent cells disrupts the homeostatic balance of immune responses, leading to a decline in immune function (2). Studies have revealed that fatty liver hemorrhagic syndrome (FLHS), triggered by excessive hepatic lipid accumulation, accounts for 40%−70% of mortality in commercially caged laying hens (3). Furthermore, the reduced immunity and antioxidant capacity of late laying hens increase their susceptibility to various diseases (4), which not only raises breeding costs but also severely impairs the economic benefits of the layer industry. This highlights the urgent need to explore effective nutritional strategies to mitigate the physiological decline of late laying hens and extend their laying cycle.

Oxidative stress, lipid metabolism disorders, and immune-inflammatory imbalance are recognized as the core physiological mechanisms underlying the decline in production performance of late laying hens (5–7). Specifically, oxidative stress damages the integrity of ovarian follicle cell membranes, accelerating follicular atresia and reducing the number of available follicles (8); it also decreases the activity of antioxidant enzymes (e.g., superoxide dismutase, SOD) in uterine tissues, thereby impairing eggshell calcification and reducing eggshell quality (9). Approximately 90% of lipid synthesis in laying hens occurs in the liver, making it the central organ for lipid metabolism and yolk precursor synthesis (10, 11). Disruption of hepatic lipid metabolism leads to excessive lipid accumulation in the liver, and inhibits the secretion of very low-density lipoprotein (VLDL); this not only causes hepatocyte damage and the development of FLHS but also serves as a major factor contributing to the decline in egg quality during the late laying period (12, 13). In addition, age-related “inflammaging” induces the excessive expression of pro-inflammatory cytokines such as interleukin-6 (IL-6) and tumor necrosis factor-α (TNF-α) (14), which exacerbates immune cell exhaustion and forms a vicious cycle that further accelerates physiological decline and organ damage in laying hens. Previous studies have confirmed that antioxidant feed additives can alleviate oxidative damage caused by heat stress in poultry (15), demonstrating that dietary intervention is a feasible and effective approach to maintaining the physiological function of laying hens (16). Thus, the identification of natural and efficient antioxidant feed additives has become a research focus in poultry nutrition.

**Figure 1:**
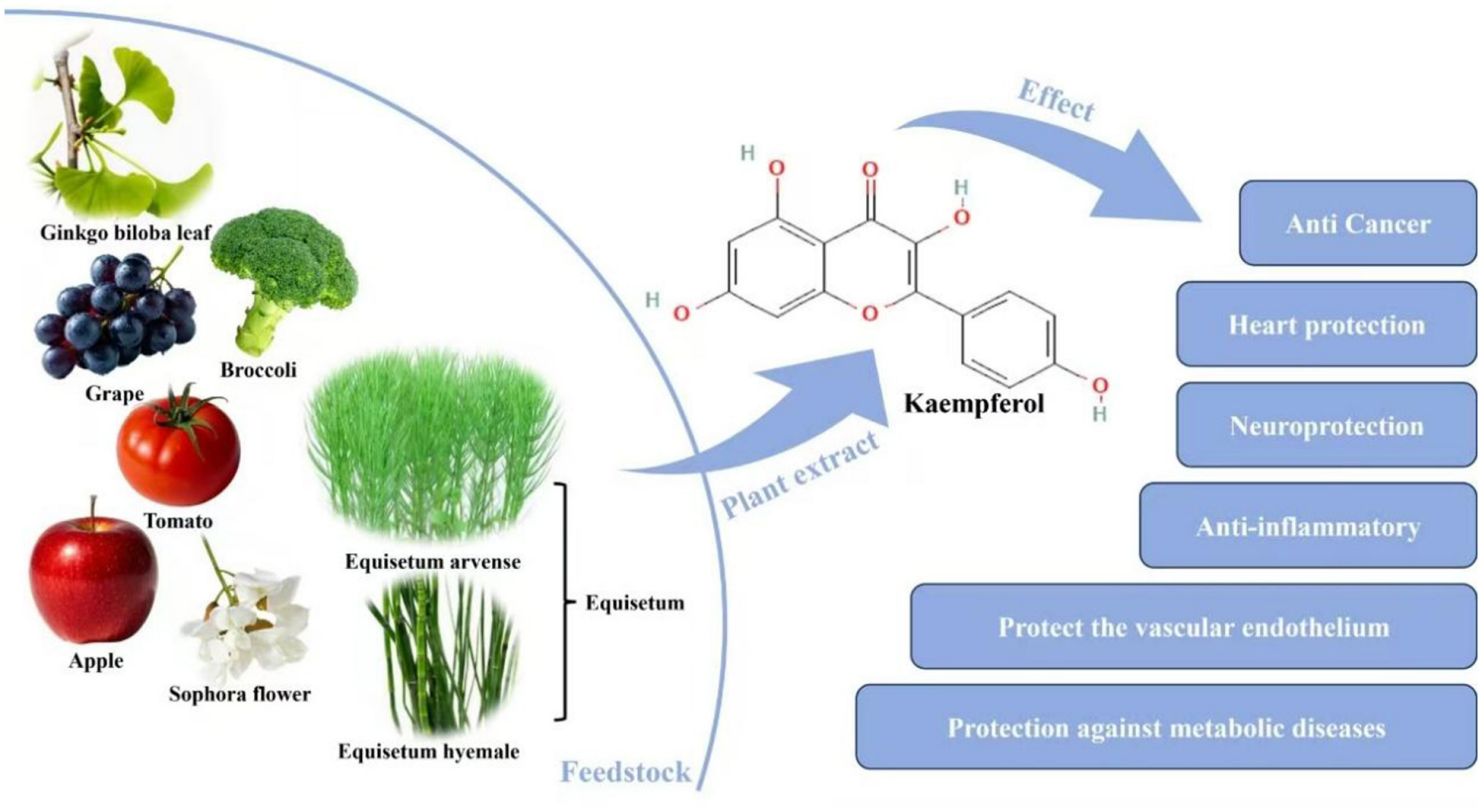
Sources and functions of kaempferol (48).

Flavonoids, a class of polyphenolic compounds widely distributed in plants, have emerged as a research hotspot in livestock and poultry nutrition due to their diverse biological activities, including antioxidant, anti-inflammatory, and metabolic regulatory effects (17). As shown in Figure 1, previous studies have shown that flavonoids can alleviate lipid accumulation in broilers by enhancing hepatic antioxidant capacity (18). In laying hens, flavonoids improve eggshell density and hardness, reducing egg breakage rates (19); they also effectively enhance yolk quality, increase yolk weight, and significantly decrease total cholesterol content in yolks (20). In weaned piglets, flavonoids balance the expression of inflammatory factors (e.g., *IL-10/IL-6*), thereby reducing the incidence of diarrhea (21). Collectively, these findings indicate that flavonoids have significant application potential for alleviating the physiological decline of livestock and poultry during the later stages of production. Kaempferol, a key member of the flavonoid family, is abundant in various fruits (e.g., strawberries, blueberries), vegetables (e.g., broccoli, spinach), and medicinal plants (22). However, systematic studies on the application of kaempferol in late laying hens are still lacking, particularly regarding its effects on hepatic lipid accumulation morphology (e.g., the degree of hepatocyte steatosis), the expression of antioxidant-related genes (e.g., *sod, GSH-PX*) in reproductive organs (uterus and ovary), and its regulatory role in immune-inflammatory balance. These knowledge gaps hinder the comprehensive evaluation of kaempferol's potential as a nutritional regulator for late laying hens.

In light of this, the present study aimed to investigate the effects of dietary kaempferol supplementation in late-stage layers. It examined whether kaempferol could improve hepatic morphological structure and alleviate lipid accumulation; evaluated its regulatory effect on the expression of antioxidant-related genes in the uterus and ovaries to assess its role in protecting reproductive function; and analyzed its impact on the levels of immune-inflammatory cytokines in serum, uterus, and ovaries to determine whether it could regulate inflammation-immune balance. This study concludes that kaempferol improves the physiological status of late-stage laying hens by regulating the antioxidant-lipid metabolism-immune inflammation axis, thereby offering a novel approach to extending their laying performance.

## 2 Materials and methods

### 2.1 Moral statement

All experimental procedures involving animals were strictly approved by the Animal Care and Use Committee of Fujian Agriculture and Forestry University (Approval ID: PZCASFAFU24046). All animal handling and sample collection were performed in accordance with the Guidelines for the Care and Use of Laboratory Animals of Fujian Agriculture and Forestry University, with the aim of minimizing animal suffering.

### 2.2 Animals and experimental design

A single-factor completely randomized design was adopted in this experiment. Two hundred eighty-eight 49-week-old Jinghong No. 1 laying hens of similar body weight and physiological status were randomly allocated to four treatment groups. Each group included 6 replicates, with 12 hens per replicate. No significant difference in egg production performance was observed among all groups before the experiment initiation. The control group received a corn-soybean meal-based basal diet. Groups I, II, and III received the same basal diet supplemented with kaempferol (KAE; purity ≥98%, supplied by Shaanxi Fullbang Biotechnology Co. Ltd., China; Batch No.: FEB221214) at doses of 0.2, 0.4, and 0.6 g/kg of feed, respectively. The gradient dose setting of kaempferol was mainly based on the optimal application dose range of quercetin, a homologous flavonol compound, in late laying hens, combined with the safe and effective dose window of flavonoids reported in poultry nutrition research (21, 23–25). The kaempferol used in this experiment was extracted from the pods of Sophora japonica, a natural and safe plant source suitable for feed additive application.

All laying hens were reared in fully enclosed cages, with a standardized feeding and management protocol implemented across all groups. Hens had free access to feed and drinking water throughout the trial period. Before the formal experiment, all hens were pre-fed with the basal diet for 1 week (at 49 weeks of age) to allow for dietary adaptation. The formal experiment commenced when the hens reached 50 weeks of age and concluded at 60 weeks of age, with a total duration of 10 weeks. At 60 weeks of age, 12 hens were randomly selected from each group (2 hens per replicate) for slaughter and sample collection, which were subsequently used for the determination of relevant indices. The nutritional levels of the basal diet were formulated in strict accordance with the NY/T33-2004 Feeding Standard for Chickens (Table 1).

**Table 1:** Composition and nutrient levels of basal diets (air-dried basis).

Ingredients (%)	Content
Corn	62.00
Soybean meal	26.00
CaHPO_4_	1.00
Limestone	8.00
*P*remix^*a*^	3.00
Total	100.00
Nutrient composition^*b*^ (%)	
Metabolic energy, ME (MJ/kg)	11.20
Crude protein, C*P*	16.80
Lysine, Lys	0.78
Methionine + Cysteine, Met + Cys	0.66
Calcium, Ca	3.70
Available phosphorus, A*P*	0.26

^*a*^The premix provides, per kg of feed, Vitamin A: 8,000 IU; Vitamin D_3_: 3,500 IU; Vitamin E: 25 mg; Vitamin K_3_: 2.5 mg; Vitamin B_1_: 4.0 mg; Vitamin B_2_: 6.0 mg; Vitamin B_6_: 5.4 mg; Vitamin B_12_: 24.0 μg; niacin: 35.0 mg; pantothenic acid: 15 mg; folic acid: 0.9 mg; biotin: 150 μg; choline chloride: 500 mg; iron: 65 mg; copper: 6.8 mg; manganese: 80 mg; zinc: 75 mg; iodine: 1.0 mg; selenium: 0.3 mg.

^*b*^Nutrient contents were calculated values.

### 2.3 Sample collection

At the 60th experimental week, two laying hens with body weights closest to the group average were selected from each group for blood collection. Approximately 10 mL of blood was drawn from the wing vein of each selected hen. The collected blood samples were first allowed to stand at room temperature for 1 h, followed by centrifugation at 3,000 rpm for 15 min. After centrifugation, the serum was carefully aspirated and stored at −20 °C until subsequent analysis. Following euthanasia of the aged laying hens, tissue samples were collected for further analysis. The liver, ovary, and uterine tissues were rapidly dissected out, immediately placed into cryopreservation tubes, snap-frozen in liquid nitrogen, and then transferred to −80 °C for long-term storage.

### 2.4 Detection of histomorphology

Liver samples were weighed according to the instructions provided with the assay kit. Physiological saline was added at a ratio of 1:9 (w/v, g:mL) to the weighed liver samples, followed by thorough homogenization to disrupt cells and release antioxidant substances. The resulting homogenate was then centrifuged at 12,000 rpm for 5 min at 4 °C, and the supernatant was collected for subsequent determinations. Serum samples were pretreated according to the kit instructions prior to analysis. The content of malondialdehyde (MDA) and the activities of total superoxide dismutase (T-SOD), catalase (CAT), glutathione peroxidase (GSH-PX), as well as the total antioxidant capacity (T-AOC) in the samples were measured. All operations were strictly performed in accordance with the manufacturer's instructions supplied with the assay kits purchased from Nanjing Jiancheng Bioengineering Institute.

### 2.5 Determination of serum and liver antioxidant-related markers

Serum was promptly separated from blood samples after collection for subsequent assays; for liver samples, the appropriate weight was measured according to the kit instructions, and physiological saline was added at a weight (g): volume (mL) ratio of 1:9 for homogenization to fully disrupt cells and release antioxidants, followed by centrifugation at 12,000 rpm (4 °C) for 5 min, with the supernatant collected for assays. The malondialdehyde (MDA) content, activities of total superoxide dismutase (T-SOD), catalase (CAT), and glutathione peroxidase (GSH-PX), as well as total antioxidant capacity (T-AOC), were measured in both serum and liver samples, with specific procedures performed in accordance with the instructions provided by the assay kits purchased from Nanjing Jiancheng Bioengineering Institute, China.

### 2.6 Determination of relative expression levels of antioxidant-related genes in the uterus and ovaries

Total RNA was extracted from uterine and ovarian tissues using the TRIzol Universal Kit (Tiangen Biotech Co., Ltd., Beijing, China) following the standard procedures: homogenization, phase separation, RNA precipitation, washing of the RNA pellet, and resuspension. The concentration and purity of the extracted RNA were quantified spectrophotometrically based on the absorbance ratios at 260 nm and 280 nm. RNA samples with an A260/A280 ratio ranging from 1.8 to 2.0 were considered to meet the experimental purity criteria, and the amount of RNA for subsequent experiments was calculated based on the measured concentration. Then, reverse transcription was performed to synthesize cDNA using a reverse transcription kit (Sangon Biotech Co., Ltd., Shanghai, China). For quantitative real-time PCR (qRT-PCR) analysis, a predefined fluorescence threshold was set for the reaction system. The cycle threshold (Ct) values of the reference gene (β*-actin*) and target genes (*cat, gsh-px, sod1, sod2)* were determined when the fluorescence signal reached this threshold. The relative expression levels of the target genes were calculated using the 2^−Δ*ΔCt*^ method, where ΔΔ*Ct* was calculated as the difference in Δ*Ct* (Δ*Ct* = Ct_targetgene_-Ctβ*-actin*) of the target gene between the experimental group and the control group. The primer sequences for qRT-PCR are listed in Table 2.

**Table 2:** Composition and nutrient levels of basal diets (air-dried basis).

Target gene	Primer sequence (5^′^ → 3^′^)	Accession no.
*β-actin*	F: CCCAGCCATGTATGTAGCCATCC	NM_205518.2
	R: AACACCATCACCAGAGTCCATCAC	
*CAT*	F: TGTTGAAAGAGGATGAACGCCAAAG	NM_001031215.2
	R: TGTCCAGCAGTGCCTGAATACG	
*GSH-PX*	F: GTGCGAGGTGAACGGGAAGG	NM_001277853.3
	R: AGATGATGTACTGCGGGTTGGTC	
*SOD1*	F: TCCTGAAGGCAAGCAGCATGG	NM_205064.2
	R: CTACTTCTGCCACTCCTCCCTTTG	
*SOD2*	F: GTCGGCGGGCAGAAGCAG	NM_204211.2
	R: TCGTAGGGCAGGTCAGGAAGAG	

### 2.7 Immune function and inflammatory cytokine testing

All assays were performed using ELISA kits from Shanghai Enzyme-Linked Biotechnology Co., Ltd., Shanghai, China, following the manufacturer's instructions. The concentrations of the following cytokines in serum as well as ovarian and uterine tissue homogenates were detected, respectively: interleukin-1β (IL-1β), gamma interferon (IFN-γ), interleukin-6 (IL-6), interleukin-4 (IL-4), interleukin-10 (IL-10), inducible nitric oxide synthase (iNOS), immunoglobulin A (IgA), immunoglobulin G (IgG), and immunoglobulin M (IgM).

### 2.8 Statistical analysis

After sorting the experimental data, one-way analysis of variance (one-way ANOVA) was performed using SPSS 26.0 statistical software, followed by Duncan's multiple comparison test. Results were expressed as “mean ± standard deviation (SD).” The thresholds for statistical significance were set at *p* < 0.05 for significance and *p* < 0.01 for high significance. 0.05 ≤ *p* ≤ 0.10 was considered a statistical trend, and *p* > 0.10 indicated no significant difference.

## 3 Results

### 3.1 Effects of kaempferol on the liver of late-laying hens

**Figure 2:**
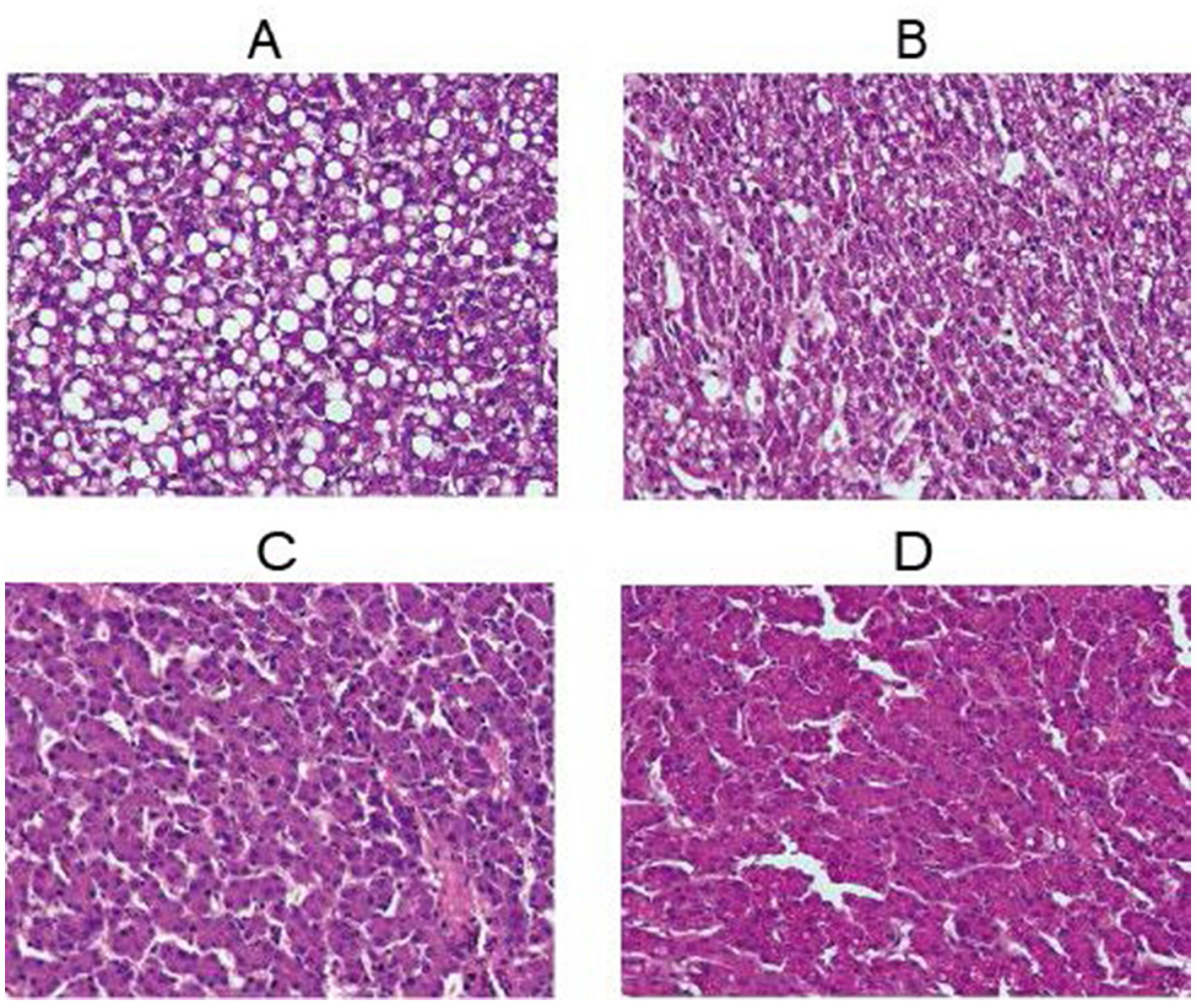
Liver section (40X) **(A)** Control Group, **(B)** Experimental Group I, **(C)** Experimental Group II, **(D)** Experimental Group III.

As shown in Figure 2, the liver tissue of the CON group exhibited obvious lipid vacuolization, with large and numerous vacuoles distributed in the hepatic lobules, indicating severe hepatic lipid deposition. In contrast, dietary kaempferol supplementation in the 0.2, 0.4, and 0.6 g/kg KAE groups resulted in a significant reduction in the hepatic vacuole area, with the hepatocellular structure being more intact and the vacuolization degree being significantly alleviated. Among the three kaempferol-supplemented groups, the 0.4 g/kg KAE group exhibited the most pronounced alleviation effect on hepatic lipid deposition, with the smallest hepatic vacuole area and the most normal hepatocellular morphology. Pathological damage of hepatic parenchyma is often the first manifestation of hepatic function impairment, and the detection of hepatic histopathological changes is the gold standard for diagnosing and evaluating the severity of hepatic lipid metabolism disorders in poultry (26).

### 3.2 The effect of kaempferol on total cholesterol in egg yolks during the late laying period

As shown in Table 3, during the 50–55-week period, there was no significant difference in egg yolk total cholesterol content among all groups (*p* > 0.05), indicating that the short-term kaempferol supplementation had no obvious effect on egg yolk cholesterol. However, during the 55–60-week period, compared with the CON group, the 0.4 and 0.6 g/kg KAE groups had significantly decreased egg yolk total cholesterol content (*p* < 0.05), while the 0.2 g/kg KAE group showed a downward trend without significant difference. For the entire experimental period (50–60 weeks), the 0.4 and 0.6 g/kg KAE groups still exhibited significantly lower egg yolk total cholesterol content than the CON group (*p* < 0.05), and there was no significant difference between the 0.4 g/kg KAE group and the 0.6 g/kg KAE group.

**Table 3:** Effect of kaempferol on total cholesterol in egg yolks during late laying hens.

Items	Groups				*p*-value
	CON	0.2 g/kg KAE	0.4 g/kg KAE	0.6 g/kg KAE	
50–55W TC (mg/g)	12.18 ± 2.83	11.02 ± 2.63	9.83 ± 2.04	9.85 ± 3.43	0.426
55–60W TC (mg/g)	11.82 ± 0.90^a^	10.40 ± 1.20^ab^	9.70 ± 1.39^b^	9.43 ± 1.60^b^	0.021
50–60W TC (mg/g)	12.00 ± 2.01^a^	10.71 ± 1.98^ab^	9.77 ± 1.67^b^	9.64 ± 2.56^b^	0.029

In the same row, different lowercase letter superscripts mean notable differences (*p* < 0.05), while different capital letters show significant differences (*p* < 0.01). Values with the same or no letters mean no significant difference (*p* > 0.05).

### 3.3 Effects of kaempferol on serum lipid metabolism in late-laying hens

**Figure 3:**
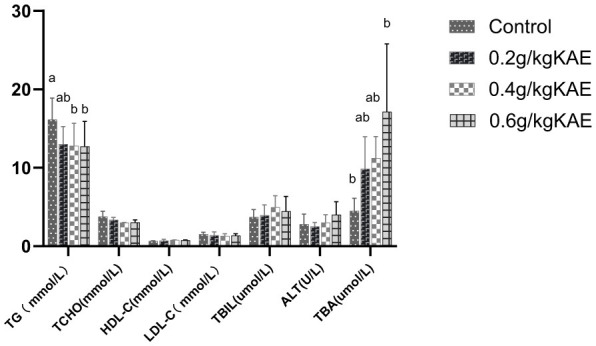
Effects of kaempferol on serum lipid metabolism in late laying hens. In the same row, different lowercase letter superscripts mean notable differences (*p* < 0.05), while different capital letters show significant differences (*p* < 0.01). Values with the same or no letters mean no significant difference (*p* > 0.05).

As shown in Figure 3, compared with the CON group, dietary supplementation with 0.4 and 0.6 g/kg KAE groups significantly reduced the serum triglyceride (TG) content of late laying hens (*p* < 0.05). In contrast, the 0.2 g/kg KAE group showed a slight decrease in serum TG content without a significant difference compared with the CON group (*p* > 0.05). There was no significant difference in serum TG content between the 0.4 g/kg KAE group and the 0.6 g/kg KAE group (*p* > 0.05), indicating that the effect of kaempferol on reducing serum TG was dose-dependent to a certain extent, and the optimal effective dose was 0.4 g/kg.

### 3.4 Effects of kaempferol on antioxidant-related indicators in serum of late-laying hens

**Figure 4:**
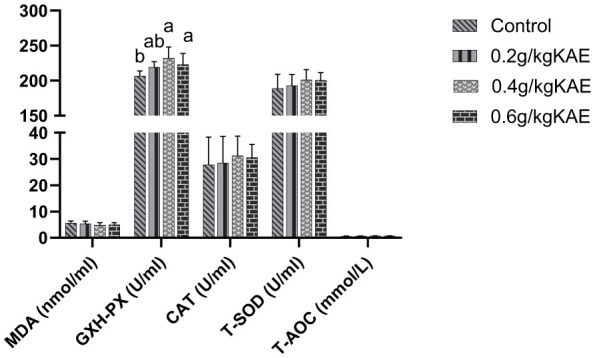
Effects of kaempferol on antioxidant-related enzymes in serum of late-laying hens. In the same row, different lowercase letter superscripts mean notable differences (*p* < 0.05), while different capital letters show significant differences (*p* < 0.01). Values with the same or no letters mean no significant difference (*p* > 0.05).

As shown in Figure 4, compared with the CON group, the 0.4 and 0.6 g/kg KAE groups significantly increased the serum glutathione peroxidase (GSH-PX) activity of late laying hens (*p* < 0.05), whereas the 0.2 g/kg KAE group had no significant effect on serum GSH-PX activity (*p* > 0.05). No significant difference was observed in serum GSH-PX activity between the 0.4 g/kg KAE group and the 0.6 g/kg KAE group (*p* > 0.05). There were no significant differences in serum T-SOD, CAT activities, MDA content, and T-AOC among all groups (*p* > 0.05).

### 3.5 Effects of kaempferol on antioxidant-related indicators in the liver of late-laying hens

As shown in Table 4, compared with the CON group, dietary kaempferol supplementation had no significant effect on hepatic MDA content, GSH-PX, CAT, and T-SOD activities in late laying hens (*p* > 0.05). However, the 0.4 g/kg KAE group showed a tendency toward increased hepatic total antioxidant capacity (T-AOC) activity (*p* = 0.072). The 0.6 g/kg KAE group also had a higher hepatic T-AOC activity than the CON group, but the increase was less pronounced than that in the 0.4 g/kg KAE group.

**Table 4:** Effects of kaempferol on antioxidant-related indicators in the liver of late-laying hens.

Items	Groups				*p*-value
	CON	0.2 g/kg KAE	0.4 g/kg KAE	0.6 g/kg KAE	
MDA (nmol/ml)	0.99 ± 0.39	0.96 ± 0.23	0.89 ± 0.28	0.87 ± 0.35	0.924
GSH-PX (U/ml)	72.62 ± 11.94	74.24 ± 7.12	87.33 ± 9.33	86.86 ± 14.84	0.096
CAT (U/ml)	7.16 ± 1.93	7.47 ± 1.88	8.80 ± 2.44	8.79 ± 2.83	0.483
T-SOD (U/ml)	189.18 ± 19.94	193.02 ± 15.87	201.25 ± 14.62	200.55 ± 10.86	0.485
T-AOCz (mmol/L)	62.59 ± 8.18	70.36 ± 9.91	80.71 ± 8.88	78.65 ± 10.99	0.072

### 3.6 Effects of kaempferol on the expression of antioxidant-related genes in the uterus of late-laying hens

As shown in Table 5, compared with the CON group, dietary kaempferol supplementation had no significant effect on the relative expression levels of *CAT, GSH-PX*, and *SOD1* genes in uterine tissue of late laying hens (*p* > 0.05). However, the 0.4 g/kg KAE group exhibited a tendency toward elevated relative expression of the *SOD2* gene in uterine tissue (*p* = 0.070), which was significantly higher than that in the 0.6 g/kg KAE group. The 0.2 g/kg KAE group also had a slightly higher *SOD2* expression than the CON group, but the difference was not significant.

**Table 5:** Effects of kaempferol on the expression of antioxidant-related genes in the uterus of late-laying hens.

Genes	Groups				*p*-value
	CON	0.2 g/kg KAE	0.4 g/kg KAE	0.6 g/kg KAE	
*CAT*	0.93 ± 0.10	0.84 ± 0.4	0.76 ± 0.4	0.10 ± 0.01	0.081
*GSH-PX*	0.77 ± 0.19	0.56 ± 0.09	0.64 ± 0.18	0.45 ± 0.15	0.128
*SOD1*	0.87 ± 0.24	0.54 ± 0.13	0.70 ± 0.43	0.65 ± 0.27	0.429
*SOD2*	0.31 ± 0.14	0.39 ± 0.11	0.67 ± 0.35	0.19 ± 0.07	0.070

### 3.7 Effects of kaempferol on the expression of antioxidant-related genes in the ovaries of late-laying hens

As shown in Table 6, compared with the CON group, dietary supplementation with 0.4 and 0.6 g/kg kaempferol significantly upregulated the relative expression level of the *SOD1* gene in ovarian tissue of late laying hens (*p* < 0.05). The 0.2 g/kg KAE group also had a higher *SOD1* expression than the CON group, but the difference was not significant (*p* > 0.05). There were no significant differences in the relative expression levels of *CAT, GSH-PX*, and *SOD2* genes in ovarian tissue among all groups (*p* > 0.05), though the 0.4 g/kg KAE group showed a tendency toward increased *GSH-PX* expression (*p* = 0.066) and a slight decrease in *SOD2* expression.

**Table 6:** Effects of kaempferol on the expression of antioxidant-related genes in the ovaries of late-laying hens.

Genes	Groups				*p*-value
	CON	0.2 g/kg KAE	0.4 g/kg KAE	0.6 g/kg KAE	
*CAT*	0.49 ± 0.21	0.45 ± 0.09	0.37 ± 0.06	0.52 ± 0.15	0.441
*GSH-PX*	0.91 ± 0.24	0.68 ± 0.28	0.61 ± 0.28	1.04 ± 0.19	0.066
*SOD1*	0.72 ± 0.06^b^	1.03 ± 0.19^ab^	1.18 ± 0.03^a^	1.28 ± 0.11^a^	0.035
*SOD2*	0.86 ± 0.12	0.70 ± 0.17	0.77 ± 0.26	0.43 ± 0.08	0.077

In the same row, different lowercase letter superscripts mean notable differences (*p* < 0.05), while different capital letters show significant differences (*p* < 0.01). Values with the same or no letters mean no significant difference (*p* > 0.05).

### 3.8 Effects of kaempferol on cytokines in serum of late-laying hens

As shown in Table 7, compared with the CON group, the 0.4 and 0.6 g/kg KAE groups significantly increased serum IL-4 levels (*p* < 0.05) and significantly decreased serum IL-1β levels (*p* < 0.05) in late laying hens. The 0.2 g/kg KAE group showed a slight increase in serum IL-4 and a slight decrease in serum IL-1β, without significant differences (*p* > 0.05). In addition, the 0.4 and 0.6 g/kg KAE groups exhibited a tendency toward decreased serum IFN-γ (*p* = 0.081) and IL-6 (*p* = 0.075) levels, and a tendency toward increased serum IL-10 levels (*p* = 0.091) compared with the CON group. Dietary kaempferol supplementation had no significant effect on serum iNOS levels among all groups (*p* > 0.05).

**Table 7:** Effects of kaempferol on cytokines in serum of late-laying hens.

Items	Groups				*p*-value
	CON	0.2 g/kg KAE	0.4 g/kg KAE	0.6 g/kg KAE	
IL-1β (pg/ml)	109.51 ± 11.30^a^	104.07 ± 11.81^ab^	91.14 ± 13.46^b^	91.99 ± 6.33^b^	0.045
IFN-γ (pg/ml)	90.42 ± 9.16	86.95 ± 9.56	76.05 ± 9.10	83.91 ± 4.60	0.081
IL-6 (pg/ml)	29.00 ± 3.09	26.96 ± 3.31	23.92 ± 3.85	24.92 ± 1.04	0.075
IL-4 (pg/ml)	142.02 ± 19.31^b^	156.78 ± 16.96^ab^	170.58 ± 12.0^a^	169.80 ± 16.99^a^	0.048
IL-10 (pg/ml)	55.60 ± 6.19	59.47 ± 6.84	66.25 ± 4.00	64.32 ± 8.92	0.091
iNOS (U/L)	7.08 ± 1.17	6.86 ± 0.29	6.38 ± 0.80	6.05 ± 1.03	0.289

In the same row, different lowercase letter superscripts mean notable differences (*p* < 0.05), while different capital letters show significant differences (*p* < 0.01). Values with the same or no letters mean no significant difference (*p* > 0.05).

### 3.9 Effects of kaempferol on serum immunoglobulin levels in late-laying hens

As shown in Table 8, compared with the CON group, dietary supplementation with 0.2, 0.4, and 0.6 g/kg kaempferol had no significant effect on serum IgM, IgG, and IgA levels in late laying hens (*p* > 0.05). Although the serum immunoglobulin levels in the kaempferol-supplemented groups were slightly higher than those in the CON group, the differences were not statistically significant, indicating that kaempferol supplementation did not affect the systemic humoral immune function of late laying hens.

**Table 8:** Effect of kaempferol on serum immunoglobulin levels in late-laying hens.

Items	Groups				*p*-value
	CON	0.2 g/kg KAE	0.4 g/kg KAE	0.6 g/kg KAE	
IgM (ug/ml)	715.08 ± 77.80	720.04 ± 40.16	787.62 ± 51.98	780.43 ± 77.00	0.184
IgG (g/l)	1,805.48 ± 256.69	2,206.94 ± 548.59	2,258.86 ± 340.81	2,219.91 ± 110.42	0.159
IgA (ug/ml)	293.99 ± 36.00	297.53 ± 34.61	330.11 ± 11.28	334.65 ± 19.41	0.061

### 3.10 Effects of kaempferol on cytokine expression in the ovaries of late-laying hens

As shown in Table 9, compared with the CON group, the 0.4 and 0.6 g/kg KAE groups significantly decreased the relative expression of *IL-1*β (*p* < 0.05) and significantly increased the relative expression of *IL-4* (*p* < 0.05) in ovarian tissue of late laying hens. In addition, the relative expression of *IL-10* in ovarian tissue was significantly higher in the 0.4 and 0.6 g/kg KAE groups than in the CON group (*p* < 0.05). The 0.2 g/kg KAE group showed a slight decrease in *IL-1*β and a slight increase in *IL-4* and *IL-10* expression in ovarian tissue, without significant differences (*p* > 0.05). There were no significant differences in the relative expression of *IFN-*γ and *IL-6* in ovarian tissue among all groups (*p* > 0.05), though the 0.4 and 0.6 g/kg KAE groups showed a downward trend in *IL-6* expression (*p* = 0.088).

**Table 9:** Effects of kaempferol on cytokine expression in the ovaries of late-laying hens.

Genes	Groups				*p*-value
	CON	0.2 g/kg KAE	0.4 g/kg KAE	0.6 g/kg KAE	
*IL-1β*	1.78 ± 0.30^a^	1.70 ± 0.48^ab^	1.12 ± 0.31^b^	1.16 ± 0.39^b^	0.048
*IFN-γ*	1.59 ± 0.67	1.59 ± 0.54	1.34 ± 0.52	1.12 ± 0.59	0.611
*IL-6*	1.82 ± 0.49	1.58 ± 0.66	1.14 ± 0.26	1.03 ± 0.30	0.088
*IL-4*	0.62 ± 0.25^b^	0.67 ± 0.29^b^	1.43 ± 0.48^a^	1.35 ± 0.37^a^	0.039
*IL-10*	0.76 ± 0.38^b^	0.96 ± 0.48^b^	1.30 ± 0.50^a^	1.24 ± 0.24^a^	0.029

In the same row, different lowercase letter superscripts mean notable differences (*p* < 0.05), while different capital letters show significant differences (*p* < 0.01). Values with the same or no letters mean no significant difference (*p* > 0.05).

### 3.11 Effects of kaempferol on cytokine expression in the uterine region of late-laying hens

As shown in Table 10, compared with the CON group, the 0.4 and 0.6 g/kg KAE groups significantly reduced the relative expression of *IL-1*β and *IL-6* (*p* < 0.05) in uterine tissue of late laying hens, and significantly increased the relative expression of *IL-4* and *IL-10* (*p* < 0.05). The 0.2 g/kg KAE group showed a slight decrease in *IL-1*β and *IL-6* expression and a slight increase in *IL-4* and *IL-10* expression in uterine tissue, without significant differences (*p* > 0.05). Dietary kaempferol supplementation had no significant effect on the relative expression of *IFN-*γ*in* uterine tissue among all groups (*p* > 0.05), though the kaempferol-supplemented groups showed a slight downward trend.

**Table 10:** Effects of kaempferol on cytokine expression in the uterine region of late-laying hens.

Genes	Groups				*p*-value
	CON	0.2g/kg KAE	0.4g/kg KAE	0.6g/kg KAE	
*IL-1β*	1.68 ± 0.39^a^	1.60 ± 0.44^ab^	0.96 ± 0.21^c^	1.03 ± 0.29^bc^	0.048
*IFN-γ*	1.30 ± 0.25	1.27 ± 0.31	0.90 ± 0.07	0.95 ± 0.25	0.150
*IL-6*	1.64 ± 0.23^a^	1.40 ± 0.11^ab^	1.02 ± 0.27^b^	1.03 ± 0.33^b^	0.041
*IL-4*	0.76 ± 0.08^b^	1.03 ± 0.33^ab^	1.40 ± 0.23^a^	1.36 ± 0.22^a^	0.029
*IL-10*	0.73 ± 0.23^b^	1.03 ± 0.28^ab^	1.02 ± 0.25^a^	1.04 ± 0.34^a^	0.049

In the same row, different lowercase letter superscripts mean notable differences (*p* < 0.05), while different capital letters show significant differences (*p* < 0.01). Values with the same or no letters mean no significant difference (*p* > 0.05).

## 4 Discussion

### 4.1 Regulatory effects of kaempferol on lipid metabolism in late-laying hens

The liver is a vital metabolic organ in laying hens, involved in a variety of key physiological processes including fatty acid synthesis, bile secretion, endogenous lipid anabolism and catabolism, yolk precursor production, and detoxification of metabolic wastes (27, 28). During the late laying period, laying hens experience a long-term high-intensity metabolic load due to sustained egg production, which easily leads to hepatic function impairment and subsequent lipid metabolism disorders. These disorders are characterized by hepatic fat vacuolization and abnormal serum lipid profiles, and can progress to fatty liver hemorrhagic syndrome (FLHS), ultimately resulting in decreased egg production and poor egg quality (29). In the present study, dietary kaempferol supplementation significantly reduced the hepatic vacuole area of late laying hens, with the effect being the most pronounced in the 0.4 g/kg KAE group, indicating that kaempferol can effectively alleviate hepatic lipid deposition and improve hepatic morphological structure. Histopathological changes in the liver, such as vacuolization and lipid droplet accumulation, are direct reflections of impaired lipid metabolism. The reduction in hepatic vacuole area observed in our study is consistent with the hepatoprotective effects of natural bioactive substances reported in previous studies. Zhu et al. (30) demonstrated that dietary supplementation with a herbaceous mixture (containing Andrographis paniculata, Silybum marianum, etc.) significantly decreased the diameter and area of hepatic vacuoles in post-peak laying hens, alleviating hepatic steatosis by reducing lipid deposition—an effect highly consistent with the lipid-lowering action of kaempferol in the present study. Xing et al. (31) further supplemented the evidence by showing that dietary porcine bile acids significantly decreased hepatic lipid droplet content (detected via Oil Red O and H&E staining) and enhanced hepatic lobule structural integrity in late laying hens. Collectively, these findings suggest that different natural bioactive substances (kaempferol, herbaceous mixtures, bile acids) may share a common “morphological repair-metabolic regulation” pathway in improving hepatic lipid deposition in late laying hens, which may be correlated with the regulation of lipolysis-related genes (e.g., lipoprotein lipase, hormone-sensitive lipase genes). Relevant studies have confirmed that kaempferol downregulates the mRNA expression of hepatic angiopoietin-like protein 3 (*ANGPTL3*), thereby relieving its inhibitory effect on lipoprotein lipase (LPL); this process accelerates the clearance of plasma triglycerides, reduces abdominal fat rate and subcutaneous fat thickness in poultry (32), which may be one of the mechanisms by which kaempferol alleviates hepatic lipid deposition in late laying hens.

In the analysis of serum lipid metabolism indices, the present study found that the 0.4 and 0.6 g/kg KAE groups had significantly decreased serum TG levels compared with the CON group, which is consistent with the above findings of alleviated hepatic lipid deposition. Previous studies have shown that the transcriptional regulatory balance between hepatic fatty acid synthesis and oxidation is a decisive factor determining triglyceride concentrations in the circulatory system and body fat accumulation (33). Sohaib et al. (34) demonstrated that dietary supplementation of flavonoid compounds (quercetin) in broilers reduces fatty acid concentrations and improves meat quality by regulating hepatic fatty acid metabolism. Combined with the results of decreased serum TG content in the present study, we speculate that kaempferol may regulate hepatic lipid metabolism in late laying hens by altering the balance of hepatic fatty acid synthesis and oxidation, thereby reducing triglyceride accumulation in the liver and serum. In addition, the 0.4 and 0.6 g/kg KAE groups significantly reduced the total cholesterol content in egg yolk, a finding that is consistent with previous research results on other flavonoids. Iskender et al. (35) compared the effects of three flavonoid compounds (hesperidin, naringin, quercetin) on laying hens and found that quercetin and hesperidin significantly decreased egg yolk cholesterol concentration, increased total egg yolk protein content, and altered lipid composition. This cholesterol-lowering effect is thought to be related to the inhibition of 3-hydroxy-3-methylglutaryl-coenzyme A (HMG-CoA) reductase activity, a key rate-limiting enzyme in hepatic cholesterol synthesis. Kaempferol, as a flavonoid compound, exhibits a similar cholesterol-lowering effect in the present study, suggesting that it may also act by inhibiting hepatic HMG-CoA reductase activity, thereby reducing cholesterol synthesis and its deposition in egg yolk.

### 4.2 The regulatory role of kaempferol in oxidative stress in late-laying hens

Aging is a complex biological process characterized by the disruption of redox homeostasis within the organism, accompanied by a sustained increase in reactive oxygen species (ROS) levels and a progressive decline in the activity of the endogenous antioxidant enzyme system (36, 37). Key antioxidant enzymes including superoxide dismutase (SOD), catalase (CAT), and glutathione peroxidase (GSH-PX), as well as non-enzymatic antioxidant molecules such as glutathione (GSH), serve as core biomarkers for evaluating oxidative stress status in avian species (38). Following the peak laying period, late laying hens experience age-related oxidative stress that induces ovarian aging and endocrine hormonal imbalances, which are major contributors to the gradual decline in production performance and egg quality (39). Therefore, enhancing the antioxidant defense and oxidative damage repair capacities of late laying hens has become a critical nutritional strategy to improve their health status and extend the effective laying cycle.

Numerous studies have demonstrated that plant-derived flavonoids possess potent antioxidant properties and can effectively alleviate oxidative stress in poultry (40, 41). Specifically, dietary supplementation with flavonoids (hesperidin, naringin, and quercetin) in laying hens has been shown to reduce malondialdehyde (MDA) concentrations—an important marker of lipid peroxidation, elevate the activities of GSH-PX, glutathione reductase (GR), glutathione S-transferase (GST), and SOD, and increase endogenous GSH levels (42), with quercetin exhibiting a more robust antioxidant effect than hesperidin and naringin. Liu et al. (24) reported that dietary quercetin supplementation improves the laying performance of hens by regulating the intestinal microflora balance and enhancing hepatic SOD activity. Furthermore, Wang et al. (43) found that low-dose quercetin upregulates the mRNA and protein expression of oxidative stress-related genes (*SOD1, CAT*, and *GSS*) in the ovaries of menopausal rats, suggesting a conserved antioxidant mechanism of flavonoids in regulating reproductive organ health across species. In addition, Dai et al. (39) showed that dietary hawthorn-leaf flavonoid supplementation reduces ovarian MDA levels and increases total antioxidant capacity (T-AOC) and GSH-PX activity in aged breeder hens, indicating enhanced free radical scavenging capacity in the reproductive system.

In the present study, we observed that the 0.4 and 0.6 g/kg KAE groups significantly increased serum GSH-PX activity; the 0.4 g/kg KAE group showed an upward trend in hepatic T-AOC content and a tendency toward increased uterine sod2 expression. Additionally, the 0.4 and 0.6 g/kg KAE groups significantly elevated ovarian *SOD1* expression—these results are consistent with those reported for other flavonoids. It can thus be inferred that kaempferol may improve the laying performance and ovarian health of laying hens by enhancing their antioxidant defense system and alleviating oxidative stress.

### 4.3 Regulatory effects of kaempferol on the immune-inflammatory balance in late-laying hens

The core function of the immune system is to maintain a delicate balance between effective pathogen defense and immune self-tolerance; dysregulation of this balance leads to chronic low-grade inflammation and impaired immune function (4). Inflammatory responses are tightly regulated by a complex network of mediators including cytokines, chemokines, adhesion molecules, and nitric oxide (NO) (44). For late laying hens, prolonged metabolic and reproductive stress disrupts immune homeostasis, inducing a state of chronic low-grade inflammation in the reproductive organs (ovary and uterus) that accelerates functional decline of these tissues. In particular, local inflammation in the ovary can disrupt follicular development and maturation, while uterine inflammation impairs eggshell calcification and formation, both of which further exacerbate the decline in laying performance and egg quality (45). Therefore, regulating the immune-inflammatory balance of the reproductive system is essential for maintaining the physiological function of late laying hens.

In the present study, the 0.4 and 0.6 g/kg KAE groups significantly increased serum IL-4 levels and decreased serum IL-1β levels (*p* < 0.05) in late laying hens, while dietary kaempferol supplementation had no significant effect on serum immunoglobulin levels (IgM, IgG, IgA) in all experimental groups (*p* > 0.05). This finding is consistent with the results of Tang et al. (46) who demonstrated that kaempferol inhibits lipopolysaccharide (LPS) and adenosine triphosphate (ATP)-induced inflammatory factor release and alleviates cardiac fibroblast inflammation by suppressing the activation of the nuclear factor-κB (*NF-*κ*B*) and protein kinase B (AKT) signaling pathways—the core inflammatory signaling cascades in eukaryotic cells. Similarly, Kong et al. (47) confirmed in a rabbit model of atherosclerosis that kaempferol reduces tumor necrosis factor-α (TNF-α) and IL-1β levels and exerts anti-vascular inflammatory effects by inhibiting the *NF-*κ*B* pathway. These mechanistic studies, combined with the results of the present study, suggest that kaempferol may exert its anti-inflammatory effect in late laying hens through the same conserved *NF-*κ*B s*ignaling pathway, by downregulating pro-inflammatory cytokines and upregulating anti-inflammatory cytokines without affecting systemic humoral immune function.

Notably, the regulatory effect of kaempferol on inflammatory cytokines was more pronounced in the reproductive organs (ovary and uterus) than in the systemic circulation. The 0.4 and 0.6 g/kg KAE groups significantly downregulated the expression of pro-inflammatory cytokines (*IL-1*β, *IL-6*) and upregulated the expression of anti-inflammatory cytokines (*IL-4, IL-10*) in both ovarian and uterine tissues (*p* < 0.05). IL-1β and IL-6 are key pro-inflammatory cytokines that trigger local inflammatory responses and tissue damage, while IL-4 and IL-10 are major anti-inflammatory cytokines that inhibit the production of pro-inflammatory factors and promote the resolution of inflammation (44). The specific regulation of inflammatory cytokine balance in the reproductive organs by kaempferol indicates a tissue-specific immune-modulatory effect, which is particularly important for late laying hens because it alleviates local inflammation in the ovary and uterus without altering systemic immune parameters—thus avoiding potential immunosuppression or immune hyperactivation. This tissue specificity may be attributed to the differential expression of flavonoid transporters and *NF-*κ*B* pathway components in the reproductive organs compared with other tissues, a hypothesis that warrants further investigation in future mechanistic studies.

In summary, kaempferol regulates the immune-inflammatory balance of late laying hens by a dual mechanism: on the one hand, it modulates the levels of pro-inflammatory and anti-inflammatory cytokines in the systemic circulation; on the other hand, it exerts a more potent and specific regulatory effect on the inflammatory response in the reproductive organs. This immune-modulatory pattern is highly beneficial for late laying hens, as it alleviates chronic low-grade inflammation in the key reproductive tissues without disrupting the systemic immune defense function, thereby maintaining the physiological function of the ovary and uterus and improving production performance.

## 5 Conclusion

Dietary supplementation of kaempferol effectively alleviates oxidative stress in laying hens by reducing hepatic lipid accumulation, serum triglyceride levels, and egg yolk cholesterol content, enhancing antioxidant enzyme (GSH-PX/CAT/SOD) activity and related antioxidant/inflammatory gene (*SOD1*/*SOD2*/*IL-4*/*IL-10*) expression, and improves immune-inflammatory balance by regulating cytokines (IL-4/IL-1β/IL-6) in serum and related cytokine genes (*IL-4*/*IL-1*β/*IL-6*) in reproductive organs, with the 0.4 g/kg KAE group exhibiting the optimal efficacy. Notably, this study innovatively reveals the tissue-specific regulatory effect of kaempferol on key organs (liver, ovary, uterus) of late laying hens and adopts a multi-dimensional design integrating histomorphology and molecular mechanisms, filling gaps in existing single-index or systemic-focused studies. In conclusion, kaempferol, as an effective plant-derived flavonoid modulating the immune-inflammatory-antioxidant axis in late-laying hens, provides important theoretical support for its development as a green feed additive to extend the laying cycle and improve hen health.

## Data Availability

The datasets presented in this study can be found in online repositories. The names of the repository/repositories and accession number(s) can be found in the article/supplementary material.
